# Comprehensive Analysis of the Polygalacturonase Gene Family and Transcriptome Screening for Candidate Genes Associated with Postharvest Softening in Atemoya

**DOI:** 10.3390/plants15121859

**Published:** 2026-06-16

**Authors:** Jinghua Huang, Luli Wang, Minmin Jing, Peiyao Chen, Xuhan Zhao, Shuailei Gu, Zhihui Chen, Jingjing Chen

**Affiliations:** 1 Key Laboratory of Tropical Fruit Biology, Ministry of Agriculture & Rural Affairs, South Subtropical Crops Research Institute, Chinese Academy of Tropical Agricultural Sciences, Zhanjiang 524091, China; huangjinghua@catas.cn (J.H.); luli_wang@catas.cn (L.W.); jingminmin@catas.cn (M.J.); chenpeiyao2026@163.com (P.C.); 15368494533@163.com (X.Z.); gushuailei@catas.cn (S.G.); chenzhihui@catas.cn (Z.C.); 2 State Key Laboratory of Tropical Crop Breeding, South Subtropical Crops Research Institute, Chinese Academy of Tropical Agricultural Sciences, Sanya 572024, China; 3 Key Laboratory of Postharvest Physiology and Technology of Tropical Horticultural Products of Hainan Province, South Subtropical Crops Research Institute, Chinese Academy of Tropical Agricultural Sciences, Zhanjiang 524091, China

**Keywords:** atemoya, polygalacturonase (PG), gene family, postharvest softening, temperature response

## Abstract

Polygalacturonase (PG) is a key enzyme in cell wall metabolism and fruit ripening. Atemoya (*Annona cherimola* Mill. × *A. squamosa* L.) is a high-value tropical fruit that undergoes rapid postharvest softening at room temperature. However, the role of the atemoya *PG* gene family in this process remains unknown. This study determined that storing atemoya at 28 °C significantly reduced fruit firmness and the total pectin content but increased water-soluble pectin (WSP) and PG activity compared to storage at 15 °C. Genome-wide identification of the *AaPG* gene family in atemoya revealed that 40 *AaPG* genes were unevenly distributed across seven chromosomes. Nineteen genes were located within six tandem duplication clusters. AaPG proteins exhibited clade-specific differences: Clades B-E contained the polysaccharide lyase family 6 (PL-6) superfamily domain, while Clade A harbored the Aspergillus niger polygalacturonase 1 (Pgu1) domain and lacked several conserved motifs. Expression profiling and reverse transcription quantitative polymerase chain reaction (RT-qPCR) showed that *AaPG19*, *AaPG21*, *AaPG23* and *AaPG24* were specifically induced at 28 °C. Subcellular localization confirmed that these four proteins were located on the plasma membrane. These findings provide insights into the evolution and temperature-dependent regulation of the *AaPG* family, identifying candidate genes responsible for the rapid softening of atemoya fruit.

## 1. Introduction

Atemoya (*Annona cherimola* Mill. × *A. squamosa* L.), an interspecific hybrid of the genus *Annona*, is predominantly cultivated in tropical regions across South Asia, East Asia, and the Americas. It is an economically significant tropical fruit crop, popular for its distinctive creamy, smooth pulp texture and rich, sweet flavor. However, it exhibits high postharvest respiration rate and undergoes rapid softening within just a few days of harvest, often accompanied by surface browning, cracking, and rot [1]. These characteristics lead to severe quality deterioration and a shortened shelf life, severely restricting its commercial value. Fruit softening, a key determinant of postharvest quality loss, is primarily driven by the disassembly and modification of cell wall structure and composition [2]. In plant cells, the cell wall, primarily composed of the middle lamella and primary wall, maintains cell shape and provides structural support. Its main structural components include cellulose, hemicellulose, pectin, glycoproteins, and lignin [3]. Notably, pectin, a major component of the middle lamella, acts as an adhesive that binds adjacent cells together [4]. Thus, the degradation of pectin directly triggers cell wall disassembly, which is a hallmark of fruit ripening and softening [4]. In the Annonaceae family, polygalacturonase (PG), cellulase (Cx), pectin methylesterase (PME), and pectate lyase (PL) exhibit high enzymatic activity during fruit softening, as observed in sugar apple (*A. squamosa* L.) [5], soursop (*A. muricata* L.) [6], and atemoya [7]. PG is particularly critical for pectin degradation [8].

PG, belonging to the glycoside hydrolase family 28 (GH28) as a core member of the pectinase family, catalyzes the hydrolysis of polygalacturonic acid, the primary structural component of pectin in plant cell walls. This enzyme is distributed not only in plants but also in bacteria and fungi [9]. A defining feature of PG is its possession of four evolutionarily conserved amino acid domains, namely SPNTDG (Domain I), GDDC (Domain II), CGPGHG (Domain III), and RIK (Domain IV) [9]. These domains form the structural basis for the catalytic activity of PG. Specifically, Domains I (NTD) and II (DD) act as the catalytic core, hydrolyzing the α-1,4-galacturonosidic bonds of pectic polysaccharides to disrupt cell wall pectin cross-links, while Domain III (H) regulates catalytic reactions for efficiency and precision, and Domain IV (RIK) enhances enzyme–pectin binding specificity via interaction with the substrate’s carboxyl group ion to ensure targeted degradation. Notably, Domain III is relatively less conserved compared to the other three domains [10].

Functionally, *PG* plays essential roles in regulating diverse physiological processes in plants, including organ morphogenesis [11], organ abscission [12,13], fruit ripening and cracking, as well as responses to biotic and abiotic stresses [14,15]. It was demonstrated that *AtPG45* functions as a polygalacturonase to regulate leaf, flower, and branch morphogenesis [16]. *OsPG1*, which encodes a polygalacturonase, modulates cell wall structure to control leaf tip necrosis in the *ltn-212* mutant [17]. In citrus, three genes (*CitPG2*, *CitPG29* and *CitPG34*) were found to be upregulated under ethylene treatment or downregulated following indole-3-acetic acid (IAA) application, indicating their potential involvement in the abscission process of citrus fruitlets [18]. Additionally, five out of 54 *PG* family members in tomato were demonstrated to be specifically or highly expressed in fruit development [19], and double knockout of *SlPG2a* and *SlPL* significantly enhanced fruit firmness and prolonged its shelf life [20].

To date, the roles of *PG* genes in fruit softening have been characterized in multiple plant species. For instance, in tomato, the mutation of the *SlPG* gene has been shown to delay fruit softening [21]. In *Actinidia arguta* (hardy kiwifruit), the *AaPG18* gene was cloned and characterized, with its expression shown to induce rapid fruit reddening, a key indicator of fruit maturity [22]. In pear, 61 *PG* gene members were identified, and it was found that transient silencing of *PbrPG6* enhances fruit firmness [23]. Similarly, *AcoPG3* acts as a key regulator of pineapple fruit softening [24]. Consistent with these findings, overexpression of the *MdPG1* gene in apple promotes cell wall degradation, induces cuticular microcracking, and accelerates premature ripening, while also modulating the expression of genes involved in water loss and ethylene biosynthesis [25]. In addition to individual gene functions, transcriptional regulation of *PGs* has also been reported. For example, in fig, *FcERF5* promotes the expression of *FcPG12* by directly binding to the GCC-box motifs in its promoter during fruit softening [26].

However, the evolutionary relationships and biological functions of the *PG* gene family (designated as *AaPG*) in atemoya remain largely unexplored. Given the critical role of *PG* in fruit softening, this study examined postharvest physiological changes in atemoya fruit stored under a low temperature (15 °C) and at room temperature (28 °C), which induce distinct softening rates. Subsequently, the *AaPG* family was systematically identified, and its chromosomal distribution, physicochemical properties, phylogeny, duplication/collinearity, conserved domains, gene structures, and cis-elements were also analyzed. To further assess their involvement in fruit softening, expression profiles were examined across different tissues and under the two temperature conditions. Additionally, subcellular localization was performed for four *AaPGs* potentially associated with softening (*AaPG19*, *AaPG21*, *AaPG23*, and *AaPG24*). Taken together, these findings establish a valuable foundation for future functional characterization of *PG* genes, particularly in the regulation of atemoya fruit softening.

## 2. Results

### 2.1. Determination of Firmness, Total Pectin Content, WSP Content and PG Activity in Atemoya Fruit

**Figure 1:**
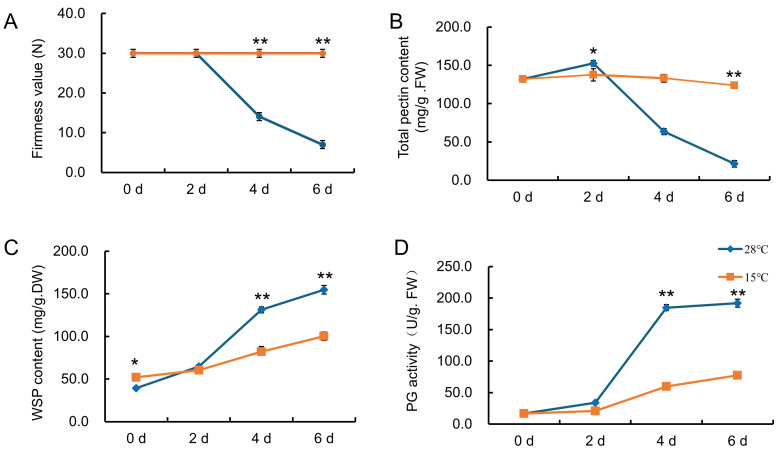
Effects of different storage temperatures (28 °C vs. 15 °C) on fruit softening-related indicators during postharvest storage. (**A**) Fruit firmness; (**B**) Total pectin content; (**C**) Water-soluble pectin (WSP) content; and (**D**) PG activity. * *p* < 0.05; ** *p* < 0.01.

Mature atemoya fruits were harvested and stored at 15 °C and 28 °C. Fruit firmness, total pectin content, water-soluble pectin (WSP) content and PG activity were determined on days 0, 2, 4 and 6 during storage (Figure 1). From day 4 onward, fruit firmness and total pectin content in the 28 °C group were markedly lower than those in the 15 °C group, while WSP content and PG activity increased significantly at 28 °C compared to 15 °C. The dynamic variation in PG activity was in accordance with the patterns of pectin metabolism and fruit softening throughout storage.

### 2.2. Genome-Wide Identification of the AaPG Gene Family in Atemoya

**Figure 2:**
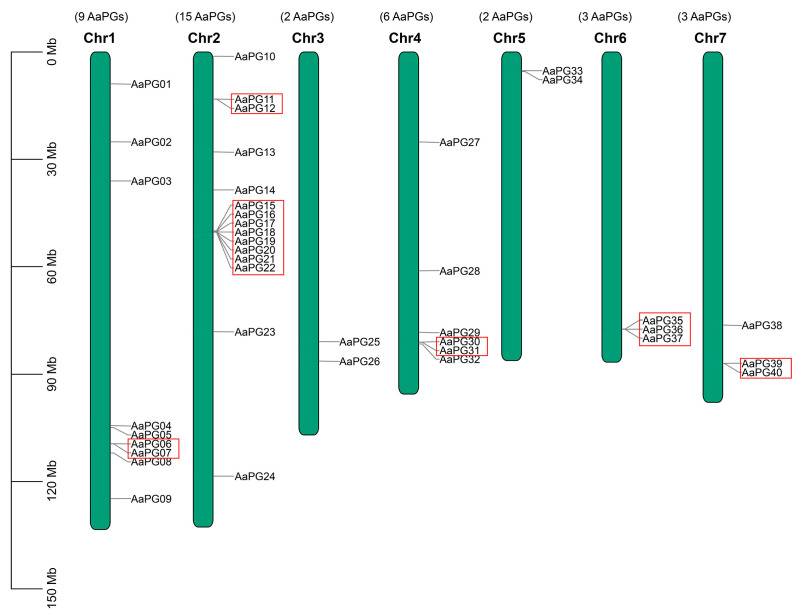
Chromosomal distribution of *AaPG* family members in the atemoya (*Annona cherimola* Mill. × *A. squamosa* L.) genome. The *AaPG* genes are mapped onto their respective chromosomes. The scale bar on the left indicates chromosome length in megabases (Mb). Tandemly duplicated gene clusters (TDGs) of *AaPG* are highlighted by red boxes. Six TDGs, designated TDG1 to TDG6, correspond to the following gene pairs: *AaPG06*–*AaPG07*, *AaPG11*–*AaPG12*, *AaPG15*–*AaPG22*, *AaPG30*–*AaPG31*, *AaPG35*–*AaPG37*, and *AaPG39*–*AaPG40*.

A total of 40 *AaPG* genes were identified in atemoya and named *AaPG01* to *AaPG40* according to their chromosomal positions (Figure 2; Table S1). The reference genome has seven pseudo-chromosomes (2*n* = 14) with a genome size of 1.5 Gb, and the scaffold N50 reaches 101.3 Mb. BUSCO analysis shows that almost 96.2–96.4% of the BUSCOs were identified at the haplotype level (Table S2). These genes were unevenly distributed across seven chromosomes. Chromosome 2 contained the largest number of genes with 15 members, followed by chromosome 1 with 9 members, while chromosomes 3 and 5 had the fewest genes with 2 members each.

### 2.3. Physicochemical Properties of AaPG Proteins

The physicochemical properties of the 40 AaPG proteins were analyzed (Table S3). The lengths of amino acid (aa) for AaPG proteins ranged from 227 to 495 aa, and their molecular weights varied from 24.06 to 55.19 kDa with an average of 44.87 kDa. The theoretical isoelectric points (pI) ranged from 5.00 to 9.74. Among them, 23 proteins exhibited basic properties with pI values above 7.0, while the remaining 17 were acidic proteins with pI values below 7.0.

Moreover, the instability index varied widely from 28.56 to 51.91. A total of 23 proteins had an instability index lower than 40, indicating biochemical stability. The aliphatic index was in the range of 71.59 to 95.74. In addition, 33 AaPG proteins exhibited negative grand average of hydropathy (GRAVY) values, indicating that the majority of AaPGs were hydrophilic. Subcellular localization prediction showed that most AaPG proteins were localized in the nucleus (16 members), followed by the chloroplast (12 members), while fewer were found in the cytoplasm (5), cell wall (5), and vacuole (2).

### 2.4. Phylogenetic Classification and Orthology Characterization of the AaPG Family

**Figure 3:**
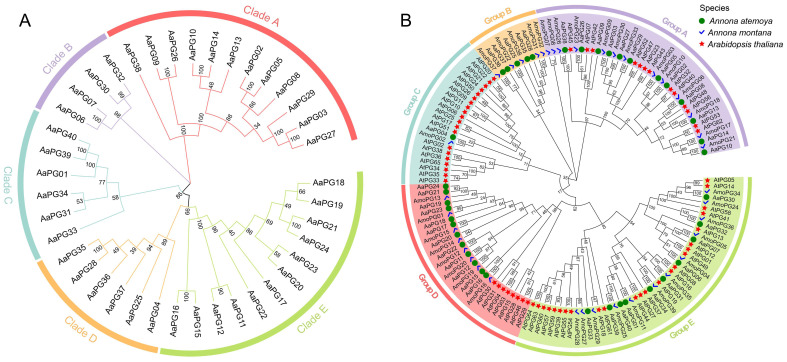
Phylogenetic analysis of PG family members. (**A**) Intraspecific phylogenetic tree of AaPGs from atemoya. (**B**) Interspecific phylogenetic tree of PG proteins from atemoya, *A. montana* (mountain soursop), and *Arabidopsis* *thaliana* (*Arabidopsis*).

An intraspecific phylogenetic tree was constructed using 40 AaPG protein sequences from atemoya (Figure 3A; Table S1). These genes were divided into five clades denoted as Clades A–E. Clades A and E were the largest, each containing 12 genes, followed by Clades C and D with six genes each, whereas Clade B contained the fewest members (four genes). An interspecific phylogenetic analysis was further performed using PG protein sequences from atemoya, *A. montana* (mountain soursop), and *Arabidopsis thaliana* (*Arabidopsis*) (Figure 3B; Tables S1, S4 and S5). All Aa*PG* genes were clustered into five groups (A–E), each containing homologs from atemoya and *Arabidopsis*. The AaPG members were distributed as Group A (12), Group D (12), Group E (10), Group B (5), and Group C (1).

Comparative orthology analysis assigned 36 of the 40 *AaPG* genes to 25 independent orthogroups (Table 1). Among these, 12 orthogroups exhibited one-to-one orthologous relationships. Nine were conserved exclusively between the two *Annona* species, and three were conserved across all three species. Paralogous expansion occurred in six orthogroups. Three expanded in both *Annona* species, two expanded only in atemoya, and one only in mountain soursop. The most prominent gene duplication events were observed in Clades 10 and 17. Clade 10 contained five atemoya paralogs (*AaPG11*, *AaPG17*, *AaPG21*, *AaPG23*, and *AaPG24*), five paralogs in mountain soursop, and two orthologs in *Arabidopsis*. Clade 17 was the largest orthogroup in the PG family, comprising four atemoya genes, six mountain soursop genes, and five *Arabidopsis* orthologs. Two orthogroups were atemoya-specific, including one single-copy gene and one paralog pair, absent in the other two species. The remaining four genes (*AaPG15*, *AaPG20*, *AaPG27*, and *AaPG37*) were not assigned to any orthogroup, possibly due to incomplete conserved domains or high sequence divergence.

### 2.5. Tandem Duplication and Selection Pressure Analysis of AaPG Genes

Six tandem duplicated gene (TDG) clusters were identified, each including two to eight genes. Among them, TDG3 is the largest, comprising eight *AaPG* genes (*AaPG15*–*AaPG22*). In total, these clusters consisted of 19 *AaPG* genes, accounting for 47.5% (19/40) of all *AaPG* members (Figure 1; Table 2). A total of 25 gene pairs within these clusters exhibited intergenic distances below 200 kb. After removing four pairs with saturated synonymous substitution rate (Ks) values (>2.0) (Table 2), the remaining 21 pairs were analyzed. The nonsynonymous/synonymous substitution rate ratios (Ka/Ks) for these 21 gene pairs ranged from 0.17 to 0.45, with an average of 0.24. All Ka/Ks values were below 0.5, and the majority (18 out of 21, 86%) were below 0.3.

### 2.6. Homology and Interspecific Collinearity Analysis of AaPG Genes

**Figure 4:**
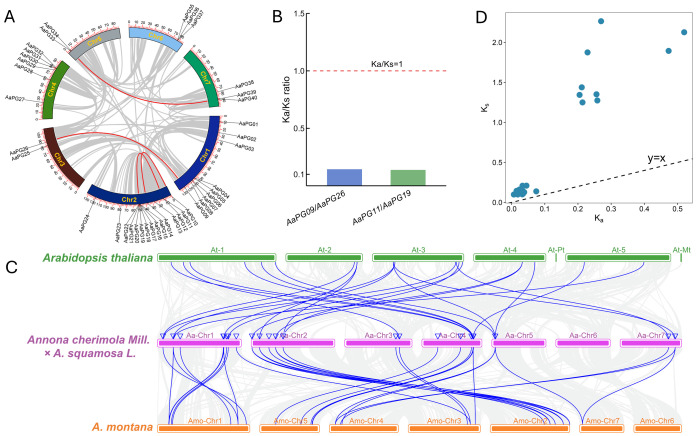
Synteny and selection pressure analysis of the *AaPG* gene family members. (**A**) Intraspecific synteny analysis of the *AaPG* gene family members in atemoya. Red lines highlight syntenic paralogous *AaPG* gene pairs, and gray lines indicate interchromosomal syntenic paralogous pairs. (**B**) Bar plot of Ka/Ks ratios for two intraspecific paralogous pairs (*AaPG09*/*AaPG26* and *AaPG11*/*AaPG19*). Dashed line indicates Ka/Ks = 1. (**C**) Interspecific synteny among *Arabidopsis thaliana* (*Arabidopsis*), *Annona cherimola* Mill. × *A. squamosa* L. (atemoya), and *A. montana* (mountain soursop). Blue lines represent orthologous *PG* gene pairs; gray lines indicate genome-wide syntenic blocks. (**D**) Scatter plot of Ka versus Ks for 34 orthologous gene pairs between atemoya and mountain soursop. Dashed line corresponds to y = x (Ka/Ks = 1).

Intraspecific synteny analysis revealed that *AaPG* genes are distributed across all seven chromosomes of atemoya. Four interchromosomal syntenic paralogous *AaPG* pairs were identified, involving 8 out of 40 *AaPG* members (20%) (Figure 4A; Table S6). Among these, two pairs (*AaPG09*/*AaPG26* and *AaPG11*/*AaPG19*) had Ks values ≤ 2.0 and were further analyzed for selection pressure. Both pairs exhibited Ka/Ks ratios below 0.3 (0.144 and 0.138, respectively), indicating purifying selection (Figure 4B). The remaining two pairs (*AaPG10*/*AaPG14* and *AaPG34*/*AaPG39*) had saturated Ks values (>2.0) and were excluded from Ka/Ks analysis.

Interspecific synteny analysis was performed among atemoya, *Arabidopsis*, and mountain soursop. A total of 32 collinear orthologous *AaPG* pairs were identified between atemoya and mountain soursop, compared with 22 orthologous pairs between atemoya and *Arabidopsis* (Figure 4C), reflecting a closer evolutionary relationship between the two *Annona* species.

Among the 32 orthologous pairs between atemoya and mountain soursop, 30 had valid Ka/Ks values. After removing two pairs with saturated Ks values (>2.0), the remaining 28 pairs were analyzed. The Ka/Ks ratios ranged from 0.07 to 0.54, with an average of 0.21. The majority of values (25 out of 28, 89%) were below 0.3, and all but one (27 out of 28, 96%) were below 0.5 (Table S7). Two distinct clusters were observed in the scatter plot: one with low Ka (<0.10) and low Ks (0.10–0.21), and another with higher Ka (0.21–0.52) and higher Ks (1.25–2.13) (Figure 4D).

### 2.7. Structural Conservation, Signal Peptide and Gene Structure Analysis of AaPG Members

**Figure 5:**
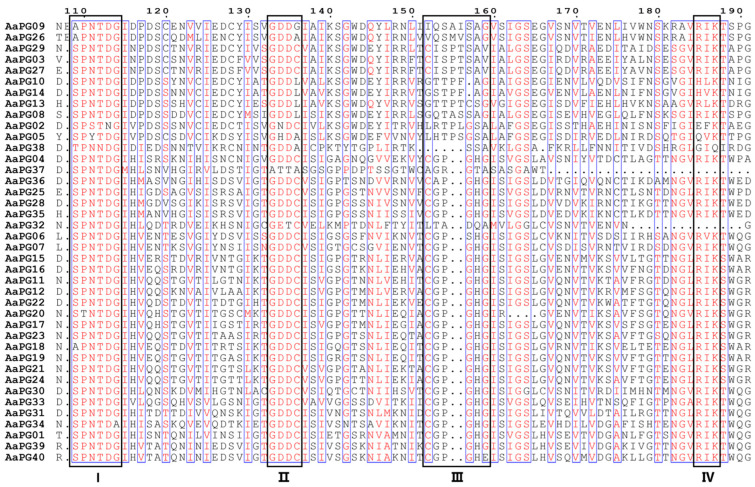
Multiple sequence alignment of AaPG family members in atemoya. Conserved amino acid residues are highlighted in red. Black boxes denote the conserved domains, corresponding to Domain I (SPNTDG), Domain II (GDDC), Domain III (CGPGHG), and Domain IV (RIK). Blue boxes indicate the highly conserved amino acid residues across all AaPG proteins in the alignment.

Multiple sequence alignment was performed to study the structural conservation of AaPG members. The alignment revealed four highly conserved functional domains (I–IV). Domains I, II, and IV were present in nearly all aligned sequences (e.g., AaPG19, AaPG23, and AaPG24). In comparison, Domain III displayed greater sequence variation, with partial or complete deletions observed in 14 AaPG members (Figure 5).

Signal peptide prediction was carried out for all AaPG proteins (Table S8). In total, signal peptides were detected in 29 out of 40 AaPG proteins (e.g., AaPG19, AaPG23, and AaPG24). Their cleavage sites spanned positions 18 to 48, and the prediction probabilities of these cleavage sites ranged from 0.5357 to 0.9825. No signal peptides were detected in the remaining 11 AaPG proteins (e.g., AaPG04 and AaPG09).

**Figure 6:**
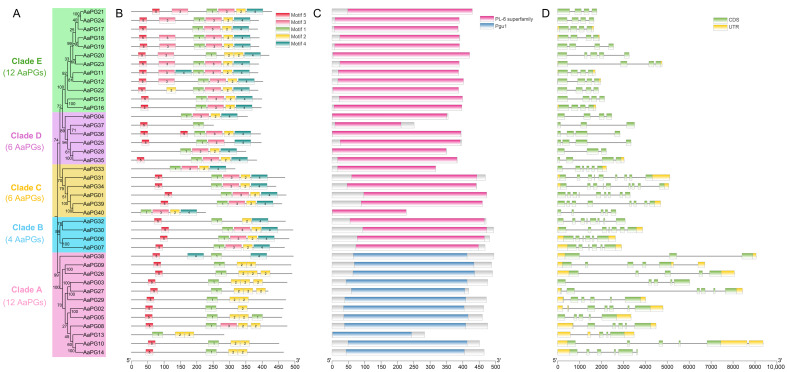
Phylogenetic relationships, conserved motifs, conserved domains, and intron–exon structures of *AaPG* gene family. (**A**) Phylogenetic tree of 40 AaPG proteins, which were divided into five clades (A–E). (**B**) Distribution of five conserved motifs (Motif 1–5). (**C**) Conserved functional domains of AaPG proteins. (**D**) Intron–exon structures of *AaPG* genes.

Conserved motif analysis revealed Clade-specific compositions (Figure 6A,B). In Clades B, C, and E with 22 proteins in total, 18 harbored all five motifs, whereas the remaining 4 lacked either motif 3 or motif 5. In contrast, all 12 members of Clade A were missing one or more motifs, predominantly motif 3 and motif 4. For Clade D (six members), two lacked motif 5, while the other four possessed the complete set of five motifs. Notably, AaPG15-AaPG22 (from the largest TDG3) were grouped into Group E, and together with AaPG11 and AaPG12 (from TDG1), as well as AaPG23 and AaPG24, all possessed all five motifs (Figure 2 and Figure 6A,B).

Conserved domain analysis showed that all members of Clades B–E harbored the PL-6 superfamily domain, whereas all members of Clade A harbored the Pgu1 superfamily domain (Figure 6C). Exon–intron structure analysis indicated that exon numbers ranged from three to nine among the 40 genes (Figure 6D). Genes belonging to A and B generally possessed full-length UTRs. Genes in Clades C, D and E typically lacked 5′ UTR, 3′ UTR, or both. At least seven genes, including *AaPG23*, *AaPG38*, and *AaPG09*, contained exceptionally long introns.

### 2.8. Analysis of Cis-Acting Elements in the Promoter Regions of AaPG Genes

**Figure 7:**
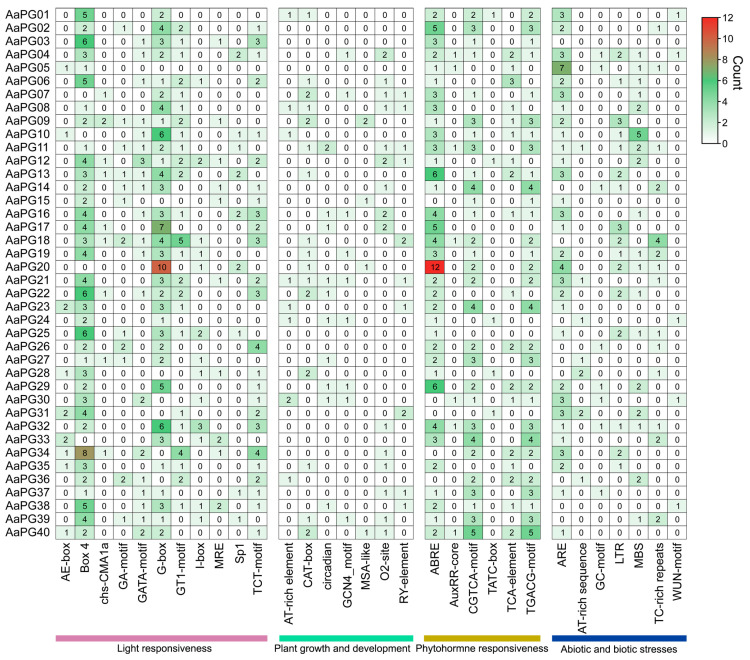
Cis-acting regulatory elements in the 2 kb upstream sequences of 40 *AaPG* genes.

The upstream 2 kb promoter regions of 40 *AaPG* genes were analyzed for cis-acting regulatory elements, which were classified into four categories: light-responsive, growth/development-related, phytohormone-responsive, and stress-responsive elements (Figure 7). A total of 31 distinct element types were identified, with highly variable distribution frequencies. The most abundant elements were Box 4 (115 occurrences), G-box (100), and ABRE (97), while TGACG-motif, TCA-element, CGTCA-motif, and ARE each appeared 64 times. In contrast, GCN4_motif (9), MSA-like (5), circadian (11), and AT-rich element (9) were rarely detected.

Among individual genes, *AaPG20* contained the highest number of elements (36 occurrences and 12 types), with exceptionally high copy numbers of ABRE (12) and G-box (10). *AaPG34*, *AaPG13*, and *AaPG17* also showed relatively high element counts, whereas *AaPG05* and *AaPG01* had the lowest overall frequencies. Specific element enrichment was observed in certain genes, such as ARE (seven in *AaPG05*), MRE (five in *AaPG10*), and LTR (two in *AaPG31*). Light-responsive elements (G-box and Box 4), an ABA-responsive element (ABRE), and MeJA-responsive elements (CGTCA-motif and TGACG-motif) were present in most *AaPG* promoters, suggesting their potential involvement in common regulatory pathways.

### 2.9. Expression Patterns of AaPG Genes in Different Tissues and Response to Temperature

**Figure 8:**
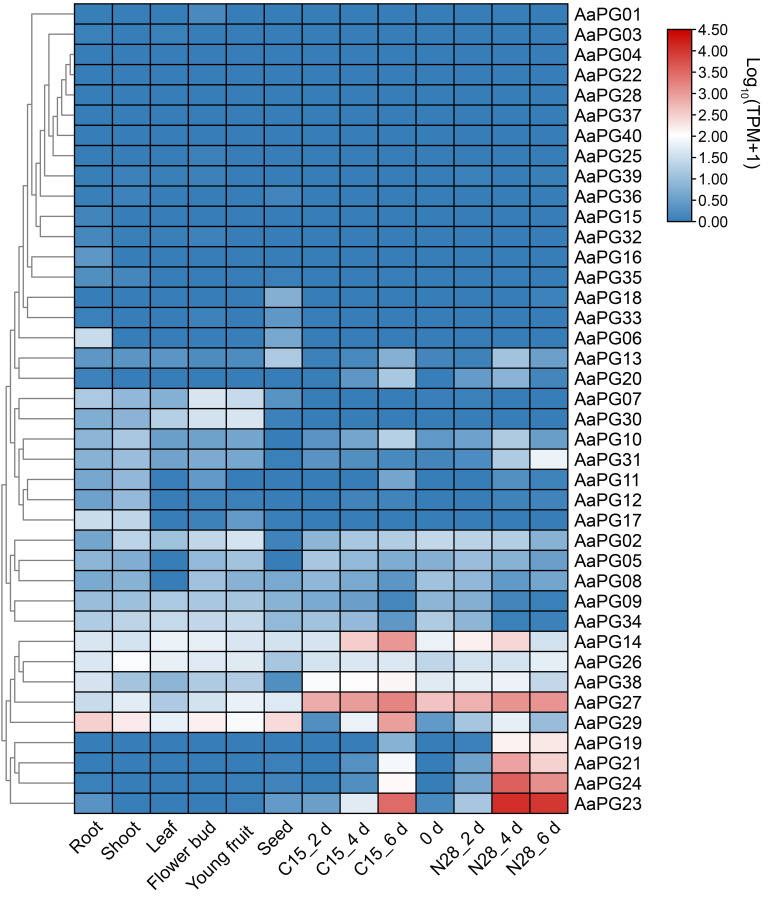
Expression patterns of the *AaPG* genes in atemoya. Tissues include root, shoot, leaf, flower bud, young fruit, and seed. Postharvest fruits were stored at 15 °C (low temperature, C15) and 28 °C (normal ripening temperature, N28) for 0, 2, 4, and 6 days. Expression values are shown as log_10_(TPM + 1) based on transcripts per million (TPM). Hierarchical clustering is based on expression similarity.

Expression patterns of 40 *AaPG* genes were examined across six atemoya tissues and in postharvest fruit stored at 15 °C or 28 °C for 0, 2, 4, and 6 days (Figure 8). About half of the 40 *AaPG* genes showed detectable expression in at least one tissue or under one temperature treatment. Under normal growth conditions, six *AaPG* genes (*AaPG09*, *AaPG34*, *AaPG14*, *AaPG26*, *AaPG27*, and *AaPG29*) showed detectable expression in vegetative and reproductive tissues.

**Figure 9:**
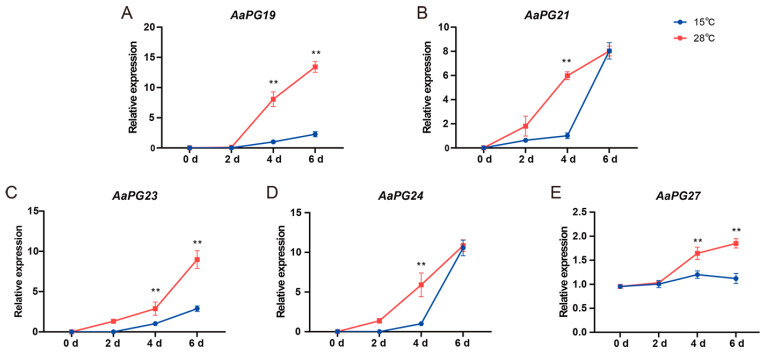
Relative expression levels of five *AaPG* genes in mature atemoya fruits stored at 15 °C or 28 °C for 0, 2, 4, and 6 days. (**A**–**E**) Relative expression levels of *AaPG19*, *AaPG21, AaPG23, AaPG24*, and *AaPG27*, respectively. ** *p* < 0.01.

Under temperature treatments, *AaPG14* and *AaPG29* were preferentially induced at 15 °C, whereas *AaPG19*, *AaPG21*, and *AaPG24* were predominantly upregulated at 28 °C. Notably, *AaPG23* was induced under both conditions but showed predominant upregulation at 28 °C, peaking at days 4 and 6. *AaPG27* was induced at both 15 °C and 28 °C. Based on their temperature responses, *AaPG19*, *AaPG21*, *AaPG23*, *AaPG24*, and *AaPG27* were selected for reverse transcription quantitative polymerase chain reaction (RT-qPCR) validation (Figure 9). RT-qPCR confirmed that *AaPG27* showed modest but significantly higher expression at 28 °C compared to 15 °C, and that *AaPG19*, *AaPG21*, *AaPG23*, and *AaPG24* were more strongly induced at 28 °C than at 15 °C.

### 2.10. Subcellular Localization of AaPG19, AaPG21, AaPG23, and AaPG24 Proteins

**Figure 10:**
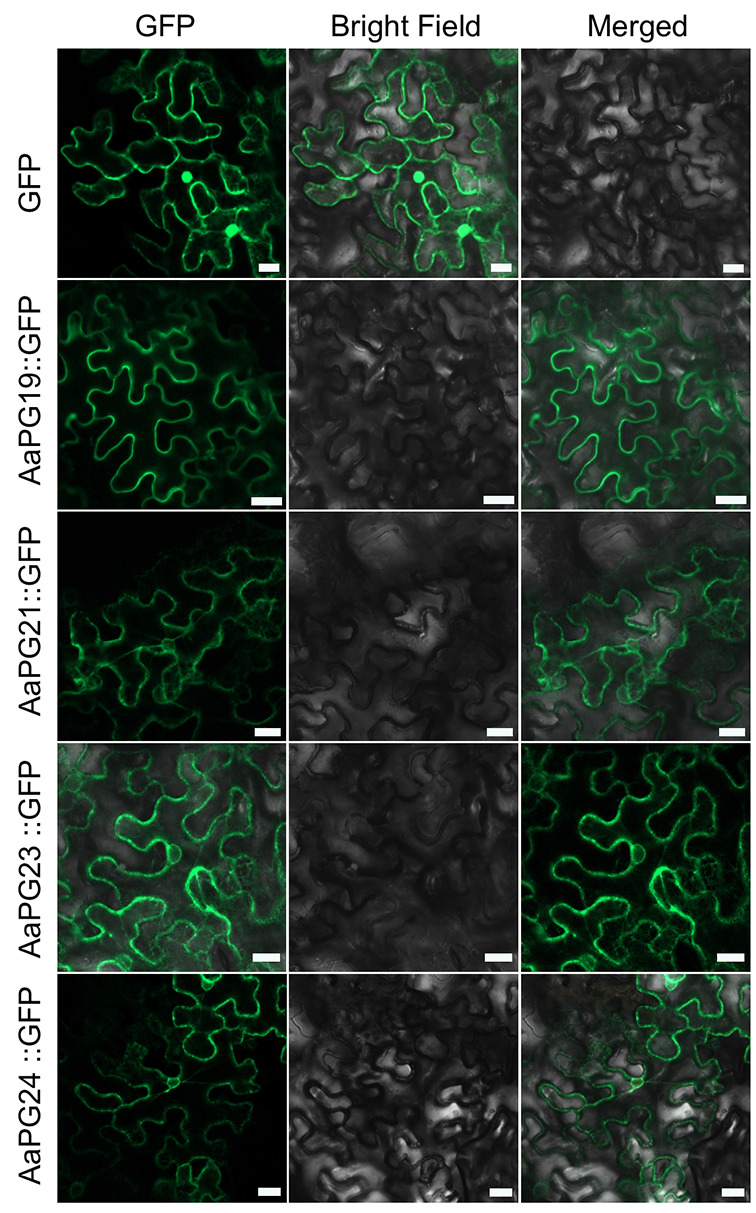
Subcellular localization of AaPG19, AaPG21, AaPG23, and AaPG24 in tobacco (*Nicotiana benthamiana*) leaves. GFP, green fluorescent protein. ‘Merged’ is the overlapped image of GFP and the bright field. Scale bars = 20 μm.

Green fluorescent protein (GFP)-fused vectors carrying *AaPG19*, *AaPG21, AaPG23, and AaPG24* were constructed and then transformed into tobacco (*Nicotiana benthamiana*) leaves. As shown in Figure 10, AaPG19--GFP, AaPG21--GFP, AaPG23-GFP and AaPG24-GFP fusion proteins overlapped with the plasma membrane contour of epidermal cells, with fluorescence signals coinciding with the cell periphery. In contrast, the free GFP control showed signals in both the cytoplasm and nucleus.

## 3. Discussion

### 3.1. Room Temperature Accelerates Fruit Softening by Enhancing PG Activity

During postharvest storage, PG activity in *Annona* fruits at room temperature (28 °C) increased significantly, accompanied by a decline in firmness and an increase in WSP content; the three processes were highly synchronized, consistent with previous reports [1,2,27]. In contrast, 15 °C treatment significantly delayed these changes, as observed in both previous studies [1,2] and the present work. This indicates that a warm environment accelerates cell wall depolymerization and softening by activating PG-mediated pectin hydrolysis [8]. Such temperature-dependent regulation of PG is common in climacteric fruits. For example, room temperature promotes PG expression and softening in peach fruit [28], while PG activity in kiwifruit is temperature-sensitive and closely correlated with firmness decline [22]. Ethylene signaling may also be involved, as its synthesis and transduction are generally affected by temperature in climacteric fruits [29,30,31]. These physiological results confirm PG as a key enzyme in *Annona* softening and provide a functional context for candidate gene screening.

### 3.2. Tandem Duplication Drives Expansion of the AaPG Family Under Strong Purifying Selection

Given on the key role of PG in softening, we performed a genome-wide identification of the *AaPG* family. Nearly half of the *AaPG* genes are located in tandem duplication clusters, a proportion significantly higher than that in grape and *Arabidopsis* [32,33], indicating that tandem duplication is the major driver of family expansion and that this expansion exhibits lineage specificity. Similar expansion patterns have been observed in cabbage, where whole-genome triplication and tandem duplications drove rapid expansion of the *BoPG* family [34]. Whole-genome duplication (WGD) also contributed to some *AaPG* members, as reported in *Arabidopsis* and rice [35], but its contribution (20%) was lower than that of tandem duplication (47.5%), further supporting the latter as the primary driver.

All duplicated *AaPG* pairs exhibited Ka/Ks < 1, and the vast majority had Ka/Ks < 0.3, indicating strong purifying selection, consistent with reports in grape [32] and *Arabidopsis* [33]. Both recent and ancient duplication events have been subject to this selective pressure [23,34]. Compared with pear PG genes (mean Ka/Ks ≈ 0.32) [23], the *AaPG* family has experienced stronger purifying selection (mean Ka/Ks = 0.21–0.24). This difference may reflect the unique biology of *Annona*. *Annona* fruit is a typical climacteric fruit that experiences extremely rapid postharvest softening, with a shelf life of only 3–5 days [29,30]. Precise cell wall remodeling is crucial for regulating the pace of softening, which may explain the stronger sequence conservation pressure on this family.

### 3.3. Structural and Cis-Regulatory Diversity Suggest Clade-Specific Functional Divergence

Although purifying selection retained core functions, phylogenetic and protein structure analyses revealed clade-specific differences within the *AaPG* family [10]. All 12 members of Clade A lacked conserved motifs 3 and 4 and carried a Pgu1 superfamily domain instead of the PL-6 domain found in other clades, whereas Clades B–E contained the typical PL-6 domain. In addition, several AaPG proteins had partial or complete deletions in the conserved Domain III region, the most variable among the four conserved domains. Evolutionary analysis of the land plant *PG* family indicated that intron loss and domain divergence were often associated with subfunctionalization or neofunctionalization after gene duplication [36,37,38]. In cabbage, the majority (76.2%) of paralogous gene pairs exhibited divergent expression patterns, also supporting this view [36].

The promoters of different *AaPG* clades differed markedly in their cis-element composition. Clade A showed enrichment of light-responsive elements but lacked fruit-ripening-associated hormone elements, consistent with a constitutive expression pattern and a possible housekeeping role. Clade B was enriched in MeJA- and SA-responsive elements, suggesting defense-related functions, while Clade D harbored metal-responsive (MRE) and stress-related (TC-rich repeats) elements, pointing to roles in metal homeostasis or stress adaptation. Within Clade E, *AaPG20* carried extremely high copies of ABRE (12) and G-box (10) but showed very low expression; conversely, *AaPG19*, *AaPG21*, *AaPG23* and *AaPG24* possessed only moderate copy numbers yet exhibited the strongest induction at specific time points (e.g., *AaPG23* >3.5). This paradox indicated that high ABRE/G-box copy numbers alone were not sufficient for strong expression; promoter context and synergistic elements were also critical, as demonstrated by synthetic promoter studies [39]. Combined with previous functional evidence for PG-mediated fruit softening [20,40], these expression and cis-element data indicated that *AaPG19*, *AaPG21*, *AaPG23* and *AaPG24* were the most likely candidate genes involved in atemoya fruit softening. Several other genes (*AaPG04*, *22*, *28*, *37*, and *40*) showed no detectable expression and might be pseudogenes.

### 3.4. Possible Roles of AaPG19/21/23/24 in Room-Temperature-Accelerated Softening

Among the multiple members of Clades B–E, the expression peaks of *AaPG19/21/23/24* coincided closely with the peak of PG enzyme activity and the increase in WSP content [6,27,29], implying that they are directly associated with the softening process. Similarly, specific PG members play key roles in softening in other climacteric fruits, such as apple *MdPG1* [25], tomato *SlPG* [20,41], pear *PbrPG6* [23], kiwifruit *AaPG18* [22], and raspberry *RiPG2* [40].

Signal peptide prediction showed that AaPG19/23/24 carry secretory signal peptides, whereas AaPG21 lacks a typical signal peptide. Nevertheless, subcellular localization results indicated that all four proteins are localized to the plasma membrane. For AaPG19/23/24, the presence of signal peptides supports their secretion to the cell wall or the plasma membrane–cell wall interface. For AaPG21, which lacks a classical signal peptide but is still localized to the plasma membrane, we speculate that it may be transported to the outer side of the plasma membrane or the cell wall region via an unconventional secretion pathway to participate in pectin degradation. Combining the expression patterns and subcellular localization results, we propose that *AaPG19*/*21*/*23*/*24* are key candidate genes involved in room temperature-accelerated softening. These findings provide targets for subsequent functional validation.

## 4. Materials and Methods

### 4.1. Plant Materials, Temperature Treatments, and Physiological Measurements

Roots, shoots, leaves, flower buds, young fruits, and seeds were collected from healthy atemoya trees. Three biological replicates were used for each tissue. For temperature treatment, mature atemoya fruits of uniform size and without visible defects were harvested and placed into incubators set at a low temperature (15 °C) and at room temperature (28 °C). Samples were taken at 0, 2, 4, and 6 days of storage, with three biological replicates per time point. All samples were frozen immediately in liquid nitrogen and stored at −80 °C.

Fruit firmness was measured as previously described [29], and results are expressed in Newtons. Total pectin and WSP were determined using commercial kits (both from Suzhou Comin, China) based on the carbazole–sulfuric acid method at 530 nm. For total pectin, fresh samples were directly extracted with dilute acid at 90 °C. For WSP, samples were pre-washed with 80% ethanol and acetone (organic solvent precipitation) to obtain crude cell wall, then extracted with acid. PG activity was assayed using a kit based on the 3,5-dinitrosalicylic acid (DNS) method (also from Suzhou Comin, China) at 540 nm. All measurements were performed on days 0, 2, 4 and 6 of storage.

### 4.2. Genome-Wide Identification of AaPG Gene Family

The atemoya genome (pseudomolecules derived from Hap2, a haplotype from *A. squamosa* or *A. cherimola*) was used for identification. Reference PG protein sequences from *Arabidopsis* and mountain soursop were downloaded from Ensembl Plants (https://plants.ensembl.org/index.html; accessed on 23 May 2025) and the NGDC database (https://ngdc.cncb.ac.cn/; accessed on 7 June 2025), respectively.

A BLASTP search was performed against the atemoya protein dataset using TBtools-II v2.467 with an E-value threshold of 1 × 10^−10^ [42]. Candidate sequences were aligned using ClustalW (https://www.genome.jp/tools-bin/clustalw; accessed on 16 May 2026). Subsequently, a hidden Markov model (HMM) search was conducted using the Pfam-A.hmm database (http://pfam.xfam.org/; accessed on 23 June 2025) with the conserved Glyco_hydro_28 domain (PF00295) as the query (E-value ≤ 1 × 10^−10^). Only sequences identified by both BLASTP and HMM were retained as initial candidates. All unique candidate proteins were further verified using InterPro [43], Pfam [44], and NCBI CD-search to confirm the presence of the core Glyco_hydro_28 domain (InterProScan ID: IPR000743; PFAM ID: PF00295).

Finally, genes carrying at least one of the four conserved domains (SPNTDG, GDDC, CGPGHG, and RIK) within the core Glyco_hydro_28 domain were designated as authentic *AaPG* members. Genes with sequences shorter than 100 amino acids or with overlong CDSs caused by genome annotation errors were excluded based on sequence length statistics and gene structure visualization. A total of 40 *AaPG* genes were identified and named *AaPG01* to *AaPG40* according to their chromosomal positions. Chromosomal distribution was visualized using TBtools-II v2.467 based on the GFF3 annotation file.

### 4.3. Physicochemical Property Analysis

The physicochemical properties of the 40 AaPG proteins were analyzed using ExPASy (https://www.expasy.org/; accessed on 23 May 2025). Parameters calculated included amino acid length, molecular weight, pI, instability index, aliphatic index, and GRAVY. Proteins with pI > 7.0 were considered basic, and those with pI < 7.0 acidic. Instability index < 40 indicated biochemical stability. GRAVY < 0 indicated hydrophilicity. Subcellular localization was predicted using WoLF PSORT (https://wolfpsort.hgc.jp/; accessed on 23 May 2025).

### 4.4. Tandem Duplication and Synteny Analysis

The genes distributed within 200 kb physical distance on the same chromosome were defined as tandem duplicated clusters. Tandem duplicated gene clusters were identified using TBtools-II v2.467 [42]. All collinear relationships and tandem duplicated loci were visualized in collinearity and positional distribution maps.

Intraspecific synteny analysis was performed for the seven atemoya chromosomes. Interspecific synteny analysis was conducted among atemoya, *Arabidopsis*, and mountain soursop using TBtools-II v2.467 [42].

The Ka, Ks, and Ka/Ks ratios for all paired genes (tandem duplicated pairs and syntenic paralogous/orthologous pairs) were calculated using TBtools-II v2.467 with the NG model. Gene pairs with Ks > 2.0 were excluded from selective pressure assessment to avoid saturated substitutions. Ka/Ks < 1 was defined as purifying selection, Ka/Ks = 1 as neutral evolution, and Ka/Ks > 1 as positive selection. Strong purifying selection was considered when Ka/Ks < 0.5.

### 4.5. Phylogenetic Tree, Conserved Motif, and Gene Structure

AaPG protein sequences were aligned with ClustalW (https://www.genome.jp/tools-bin/clustalw; accessed on 16 May 2026). Multiple sequence alignment of AaPG proteins was visualized using ESPript 3.2 (https://espript.ibcp.fr/ESPript/cgi-bin/ESPript.cgi). Intraspecific phylogenetic tree (AaPGs) and interspecific tree (including AaPGs, mountain soursop AmoPGs, and Arabidopsis AtPGs) were constructed using the neighbor-joining method with 1000 bootstrap replicates in MEGA 12.0. For *Arabidopsis*, 66 AtPG protein sequences were retrieved from TAIR10, excluding At1g23470 (annotated as a non-functional pseudogene) (Table S5). Trees were visualized using Evolview 3.0 (https://www.evolgenius.info/evolview-v3/).

Conserved motifs were identified using MEME (http://meme-suite.org/tools/meme; accessed on 17 May 2026) with a maximum of 5 motifs and default parameters. Conserved domains were verified using NCBI CD-search. Exon, intron, and UTR information was extracted from the atemoya GFF3 annotation. An integrated map of phylogeny, conserved motifs, functional domains and gene structure was generated using TBtools-II v2.467 [42]. Signal peptide prediction was performed using SignalP 6.0 (https://services.healthtech.dtu.dk/services/SignalP-6.0/).

### 4.6. Promoter Cis-Acting Element Analysis

The upstream 2 kb promoter regions of all 40 *AaPG* genes were extracted from the atemoya genome. Cis-acting elements were predicted using the PlantCARE database (http://bioinformatics.psb.ugent.be/webtools/plantcare/html/; accessed on 18 May 2026). The identified elements were classified into four categories: light-responsive, growth/development-related, phytohormone-responsive, and stress-responsive elements.

### 4.7. Expression Analysis Based on Transcriptome Data

Two RNA-seq datasets previously generated in the lab were reanalyzed (accession number: PRJCA064963) [27,45]. The first dataset included six tissues: root, shoot, leaf, flower bud, young fruit, and seed. The second dataset came from postharvest fruits stored at 15 °C or 28 °C for 0, 2, 4, and 6 days. Raw read counts were normalized to transcripts per million (TPM) to obtain normalized expression levels. The clean reads were quantified at the transcript level using Salmon (v1.10.0) in quasi-mapping mode. A decoy-aware index was built using the atemoya reference genome (Hap2) and annotation file (GFF3). The expression levels were estimated as TPM, which accounts for both sequencing depth and effective gene length. For heatmap visualization, a log_10_(TPM + 1) transformation was applied. Heatmaps were drawn using the heatmaps package in TBtools-II v2.467 [42].

### 4.8. RNA Extraction and RT-qPCR

Total RNA was extracted using the RNAprep Pure Plant Plus Kit (Tiangen, Beijing, China). First-strand cDNA was synthesized with the TransScript^®^ One-Step gDNA Removal and cDNA Synthesis SuperMix (Transgen, Beijing, China). RT-qPCR was performed on an Applied Biosystems QuantStudio^™^ 6 Flex system using Hieff UNICON^®^ Universal Blue qPCR SYBR Green Master Mix (Yeasen, Shanghai, China). Each 10 μL reaction contained 5.0 μL of SYBR Green Master Mix, 0.2 μL of each primer (10 μM), 1.0 μL of diluted cDNA (1:5), and 3.6 μL of RNase-free water. The thermal cycling conditions were 95 °C for 2 min, followed by 40 cycles of 95 °C for 10 s and 60 °C for 30 s. Three technical replicates were performed for each sample. *AcActin* was used as the reference gene [46], and the relative expression of genes was calculated using the 2^−ΔΔCT^ method [47]. All gene-specific primers used in this experiment are listed in Table S9.

### 4.9. Subcellular Localization Assays in Tobacco

The coding sequences (without stop codons) of candidate *AaPG* genes were amplified from atemoya fruit cDNA and individually cloned into the pYL1300-GFP vector under the control of the CaMV 35S promoter. Each recombinant construct, together with the empty vector control (35S::GFP), was transformed into *Agrobacterium tumefaciens* strain GV3101. The transformed agrobacteria were infiltrated into leaves of 4-week-old *Nicotiana benthamiana* plants. After 72 h of growth at 22 °C under a 16 h light/8 h dark photoperiod, GFP fluorescence in leaf disks was observed using a Zeiss LSM780 confocal microscope (Zeiss, Jena, Germany). GFP signals were excited at 488 nm and detected at 500–530 nm. Primers used for vector construction are listed in Table S9.

## 5. Conclusions

In summary, this study provides a comprehensive characterization of the polygalacturonase gene family in atemoya. Room temperature (28 °C) accelerates fruit softening by enhancing PG activity and pectin solubilization, while low temperature (15 °C) delays these processes. The *AaPG* family comprises 40 members, expanded primarily through tandem duplication under purifying selection. The proteins form five clades, with Clade A carrying the Pgu1 domain and lacking several motifs, while Clades B–E harbor the PL-6 domain, suggesting subfunctionalization. Promoters are enriched with ABA- and light-responsive elements. Expression profiling revealed a room-temperature-induced module (*AaPG19*, *AaPG21*, *AaPG23*, and *AaPG24*) and two low-temperature-preferential genes (*AaPG14* and *AaPG29*). High expression of the four room-temperature-induced genes correlates with increased PG activity and softening, and subcellular localization confirmed their plasma membrane localization. Collectively, these findings provide insights into the evolution and regulatory mechanisms of the *AaPG* gene family for functional validation of candidate genes involved in postharvest softening. Future studies should focus on genetic transformation or gene editing to confirm their precise roles in temperature-dependent softening.

## Figures and Tables

**Table 1:** Orthology classification of *PG* genes among atemoya, *Arabidopsis thaliana* (*Arabidopsis*), and *A. montana* (mountain soursop).

Clade	Gene ID (atemoya)	Gene ID (*Arabidopsis*)	Gene ID (mountain soursop)	Orthology Classification
1	*AaPG01*	-	*AmoPG11*	One-to-one ortholog between two *Annona* species
2	*AaPG02*, *AaPG05*	*AtPG40*	*AmoPG03*, *AmoPG10*	Paralogous expansion in both *Annona* species
3	*AaPG03*	-	*AmoPG09*, *AmoPG30*	Paralogous expansion in *Annona*
4	*AaPG04*	-	*AmoPG02*	One-to-one ortholog between two *Annona* species
5	*AaPG06*	*AtPG01*	*AmoPG04*	One-to-one ortholog among three species
6	*AaPG07*	*AtPG12*	*AmoPG05*	One-to-one ortholog among three species
7	*AaPG08*	-	-	Species-specific gene (atemoya)
8	*AaPG09*, *AaPG26*	*AtPG07*, *AtPG42*	*AmoPG23*	Paralogous expansion in atemoya
9	*AaPG10*	-	*AmoPG21*	One-to-one ortholog between two *Annona* species
10	*AaPG11*, *AaPG17*, *AaPG21*, *AaPG23*, *AaPG24*	*AtPG31*, *AtPG46*	*AmoPG01*, *AmoPG13*, *AmoPG14*, *AmoPG15*, *AmoPG20*	Paralogous expansion in both *Annona* species
11	*AaPG12*	-	*AmoPG19*	One-to-one ortholog between two *Annona* species
12	*AaPG13*	*AtPG56*	*AmoPG18*	One-to-one ortholog among three species
13	*AaPG14*	*AtPG53*	*AmoPG17*	One-to-one ortholog among three species
14	*AaPG16*	-	*AmoPG16*	One-to-one ortholog between two *Annona* species
15	*AaPG18*, *AaPG19*,	-	-	Species-specific paralog (atemoya)
16	*AaPG22*	-	*AmoPG12*	One-to-one ortholog between two *Annona* species
17	*AaPG25*, *AaPG28*, *AaPG35*, *AaPG36*	*AtPG33*, *AtPG34*, *AtPG35*, *AtPG51*, *AtPG65*	*AmoPG07*, *AmoPG22*, *AmoPG31*, *AmoPG32*, *AmoPG37*, *AmoPG38*	Paralogous expansion in both *Annona* species
18	*AaPG29*	-	*AmoPG33*	One-to-one ortholog between two *Annona* species
19	*AaPG30*	*AtPG05*	*AmoPG34*	One-to-one ortholog among three species
20	*AaPG31*	*AtPG08*	*AmoPG35*	One-to-one ortholog among three species
21	*AaPG32*	*AtPG41*	*AmoPG36*	One-to-one ortholog among three species
22	*AaPG33*	-	-	Species-specific gene (atemoya)
23	*AaPG34*	*AtPG19*	*AmoPG39*	One-to-one ortholog among three species
24	*AaPG38*	*AtPG45*	*AmoPG26*	One-to-one ortholog among three species
25	*AaPG39*, *AaPG40*	*AtPG37*	*AmoPG25*	Paralogous expansion in atemoya

**Table 2:** Summary of tandem duplicated *AaPG* gene pairs in atemoya.

No.	TDG Cluster Name	Gene Pairs	Physical Distance (kb)	Ka	Ks	Ka/Ks
1	TDG1	*AaPG06*-*AaPG07*	31.11	0.31	2.43	-
2	TDG2	*AaPG11*-*AaPG12*	8.30	0.23	1.33	0.17
3	TDG3	*AaPG15*-*AaPG16*	104.58	0.12	0.44	0.27
4	TDG3	*AaPG16*-*AaPG17*	164.12	0.36	2.15	-
5	TDG3	*AaPG16*-*AaPG18*	193.32	0.35	2.91	-
6	TDG3	*AaPG17*-*AaPG18*	27.56	0.25	1.21	0.21
7	TDG3	*AaPG17*-*AaPG19*	33.22	0.22	0.98	0.22
8	TDG3	*AaPG17*-*AaPG20*	49.38	0.28	1.16	0.24
9	TDG3	*AaPG17*-*AaPG21*	66.76	0.20	1.00	0.20
10	TDG3	*AaPG17*-*AaPG22*	79.48	0.28	1.57	0.18
11	TDG3	*AaPG18*-*AaPG19*	3.76	0.15	0.64	0.23
12	TDG3	*AaPG18*-*AaPG20*	19.91	0.26	0.93	0.29
13	TDG3	*AaPG18*-*AaPG21*	37.30	0.17	0.80	0.22
14	TDG3	*AaPG18*-*AaPG22*	50.01	0.33	1.63	0.20
15	TDG3	*AaPG19*-*AaPG20*	13.61	0.22	0.82	0.27
16	TDG3	*AaPG19*-*AaPG21*	30.99	0.14	0.63	0.22
17	TDG3	*AaPG19*-*AaPG22*	43.71	0.30	1.78	0.17
18	TDG3	*AaPG20*-*AaPG21*	14.12	0.22	0.67	0.34
19	TDG3	*AaPG20*-*AaPG22*	26.84	0.34	1.80	0.19
20	TDG3	*AaPG21*-*AaPG22*	10.93	0.29	1.70	0.17
21	TDG4	*AaPG30*-*AaPG31*	31.31	0.66	4.66	-
22	TDG5	*AaPG35*-*AaPG36*	79.04	0.26	1.20	0.22
23	TDG5	*AaPG35*-*AaPG37*	85.92	0.41	0.91	0.45
24	TDG5	*AaPG36*-*AaPG37*	4.04	0.31	0.77	0.40
25	TDG6	*AaPG39*-*AaPG40*	36.75	0.06	0.34	0.18

Note: All gene pairs presented here have an intergenic physical distance of less than 200 kb. Gene pairs with Ks > 2.0 (Nos. 1, 4, 5, and 21) were considered saturated and excluded from Ka/Ks analysis (indicated by “-”).

## Data Availability

The RNA-seq data are available at the NGDC (https://ngdc.cncb.ac.cn/) under accession number PRJCA064963.

## Supplementary Materials

The following supporting information can be downloaded at https://www.mdpi.com/article/10.3390/plants15121859/s1, Table S1: Protein sequences of identified AaPGs in atemoya. Table S2: Genome assembly and annotation features of atemoya. Table S3: Nomenclature and protein properties of AaPGs in atemoya. Table S4: Protein sequences of identified AmoPGs in mountain soursop. Table S5: Protein sequences of AtPGs in *Arabidopsis*. Table S6: The Ka/Ks ratio of paralogous genes in the *AaPG* gene family. Table S7: Selection pressure analysis of homologous *PG* genes between atemoya and mountain soursop. Table S8: Signal peptide prediction of AaPG family proteins. Table S9: Primers used for RT-qPCR analysis of *AaPGs*.
